# Balancing perfectionism with expansion in community CPR training

**DOI:** 10.1016/j.resplu.2026.101301

**Published:** 2026-03-24

**Authors:** Jeremy Pallas, Mark Miller, Shaun Hicks, Phillip Newton, Ginger Chu, John Paul Smiles, Michael Zhang

**Affiliations:** a School of Nursing and Midwifery, University of Newcastle, Newcastle, Australia; b Emergency Department, John Hunter Hospital, Newcastle, Australia; c Hunter Heart Safe, Newcastle, Australia; d Nephrology Department, John Hunter Hospital, Newcastle, Australia; e Asthma and Breathing Program, Hunter Medical Research Institute, Newcastle, Australia

Dear Editors of Resuscitation Plus,

We write to provide perspective on our paper published in January.^1^ In this study of a generational cardiopulmonary resuscitation (CPR) training intervention, the Laerdal QCPR® score was incorporated into a post-training assessment tool with a passing QCPR threshold of ≥50. During peer review, it was appropriately observed that a QCPR score of 50 is unlikely to represent “high-quality” CPR. As described, our chosen threshold reflected investigator consensus regarding a pragmatic minimum competency floor for the community rescuer, rather than an attempt to define high performance. Nevertheless, this feedback invites contemplation of how QCPR scores from our dataset may be interpreted.

While Laerdal describes the composition of their proprietary QCPR scoring algorithm,^2^ there is no universally accepted framework linking specific scores to accepted qualitative descriptors. Where on one hand, the Resuscitation Quality Improvement Program® (co-developed by Laerdal and the AHA) proposes an adequacy threshold of 75% with a focus on full CPR provided by healthcare professionals,^3^ Cortegiani identifies the same QCPR threshold of ≥75 as an indicator of ‘advanced’ CPR performance.^4^ Limitations notwithstanding, this alternative benchmark provides a useful secondary lens through which to examine our findings in a community based, compression-only intervention. Fig. 1 is provided to graphically represent the QCPR scores of all participants in our study with added colour coding to reflect the different performance bands.

**Fig. 1:**
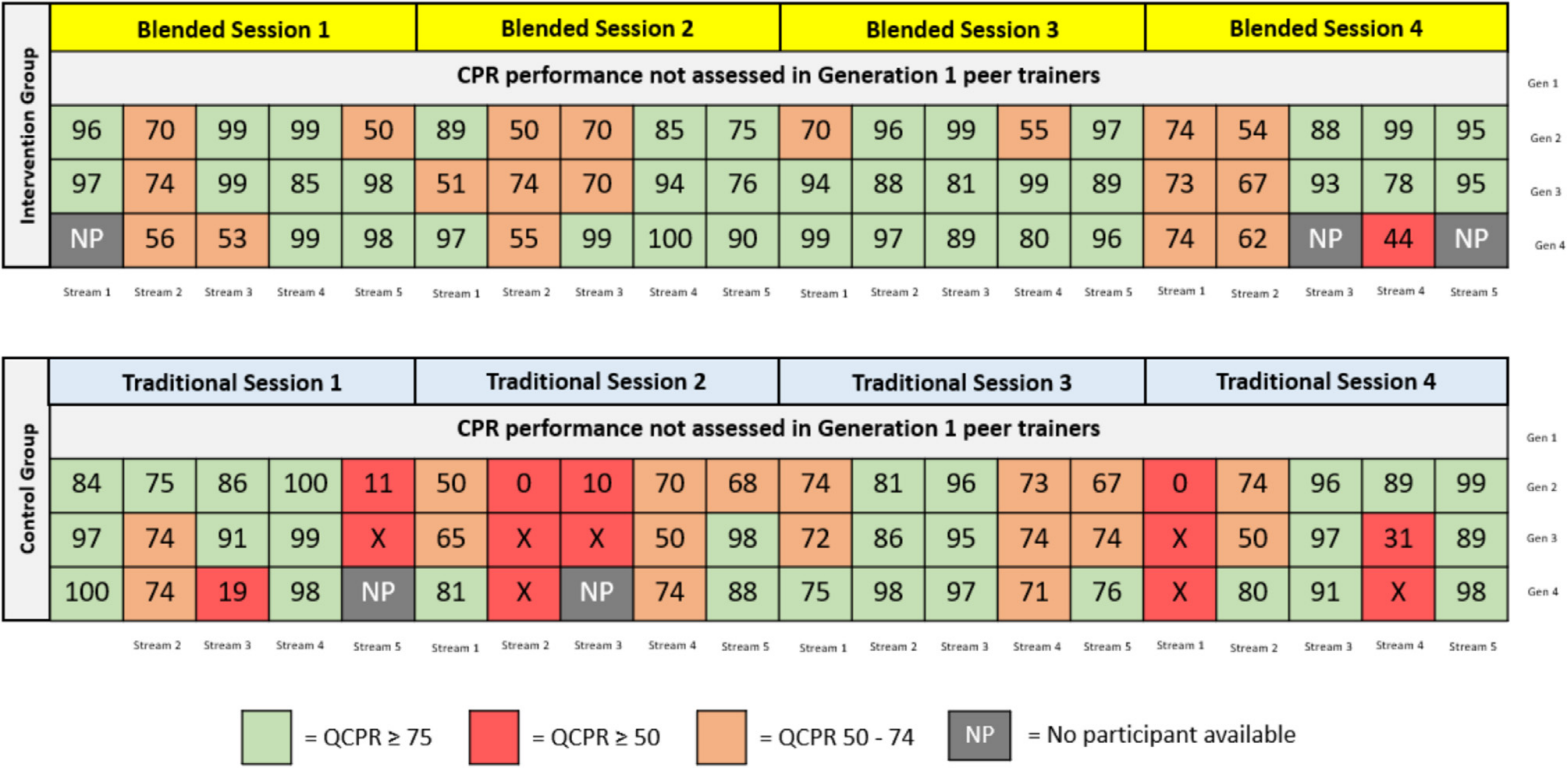
**QCPR data from Pallas et al. (2026) contextualised for additional performance bands**.

Applying a ≥75 threshold to our data yields important observations. In our cascading model, each generation was trained before teaching the next, with progression contingent upon passing an intergenerational assessment. We hypothesised that instructional fidelity—and therefore CPR performance—would decline across generations, and that participants performing poorly would be unlikely to train subsequent learners effectively.

Somewhat unexpectedly, QCPR scores did not deteriorate across generations and in fact generally improved. Fourteen participants achieved QCPR scores ≥75 despite having first-generation trainers who scored below this performance threshold. Although the scale of our study precludes causal inference, these findings challenge the assumption that trainer performance rigidly determines downstream trainee competence within cascading models.

Importantly, had a ≥75 threshold been adopted as the required passing standard, intergenerational progression would have ceased earlier in multiple chains. Within our cohort, this would have resulted in approximately 20% fewer trained participants (*n* = 32). In practical terms, a higher performance bar would have materially reduced community penetration of CPR training.

While the pursuit of optimal CPR quality is unquestionably important, competency thresholds are not value-neutral; they shape how widely life-saving skills disseminate. In communities where fewer than half of out-of-hospital cardiac arrests receive bystander CPR,^5^ restricting progression to only those achieving near-advanced competency metrics risks privileging performance purity over public health impact. A strategy that modestly lowers the performance ceiling but substantially increases responder saturation may reasonably be expected to yield greater aggregate survival benefit than one that produces fewer, technically superior responders.

We therefore contend that in scalable community training models, the balance between technical optimisation and maximal uptake must be made explicit. On this basis, we further urge caution in a delicate balance that may see technical perfection framed as a barrier to entry for would be community rescuers.

## CRediT authorship contribution statement

**Jeremy Pallas:** Conceptualization, Writing – original draft, Writing – review & editing. **Mark Miller:** Conceptualization, Writing – review & editing. **Shaun Hicks:** Writing – review & editing. **Phillip Newton:** Writing – review & editing. **Ginger Chu:** Writing – review & editing. **John Paul Smiles:** Writing – review & editing. **Michael Zhang:** Writing – review & editing.

## Ethics approval

Not applicable (letter to the editor).

## Funding

This trial has been funded by a philanthropic grant from the Jack Murphy Memorial Society, which was administered by the Hunter Medical Research Institute (HMRI). For part of the duration of this trial, the Principal Investigator (Jeremy Pallas) was supported by the Australian Governments Research Training Program (RTP) Scholarship – to support a Higher Degree Research (HDR) program.

## Declaration of competing interest

The authors declare no conflicts of interest.
